# Optimising collection of footprint evidence from crime scenes and suspects

**DOI:** 10.1186/1757-1146-4-S1-P3

**Published:** 2011-05-20

**Authors:** Paul J Bennett

**Affiliations:** 1 Queensland University of Technology, School of Public Health, Brisbane, Qld, Australia

## 

Analysis of either footprints or footwear impressions which have been recovered from a crime scene is a well known and well accepted part of forensic investigation. When this evidence is obtained by investigating officers, comparative analysis to a suspect’s evidence may be undertaken. This can be done either by the detectives or in some cases, podiatrists with experience in forensic analysis. Frequently asked questions of a podiatrist include; “What additional information should be collected from a suspect (for the purposes of comparison), and how should it be collected?” This paper explores the answers to these and related questions based on 20 years of practical experience in the field of crime scene analysis as it relates to podiatry and forensics. Elements of normal and abnormal foot function are explored and used to explain the high degree of variability in wear patterns produced by the interaction of the foot and footwear. Based on this understanding the potential for identifying unique features of the user and correlating this to footwear evidence becomes apparent. Standard protocols adopted by podiatrists allow for more precise, reliable, and valid results to be obtained from their analysis. Complex data sets are now being obtained by investigating officers and, in collaboration with the podiatrist; higher quality conclusions are being achieved. This presentation details the results of investigations which have used standard protocols to collect and analyse footwear and suspects of recent major crimes.

